# Intragroup and intragenomic conflict over chemical defense against predators

**DOI:** 10.1002/ece3.3926

**Published:** 2018-02-19

**Authors:** Rebekah Best, Graeme D. Ruxton, Andy Gardner

**Affiliations:** ^1^ School of Biology University of St Andrews St Andrews UK

**Keywords:** automimicry, cochineal, genomic imprinting, inclusive fitness, kin selection, predation

## Abstract

Insects are often chemically defended against predators. There is considerable evidence for a group‐beneficial element to their defenses, and an associated potential for individuals to curtail their own investment in costly defense while benefitting from the investments of others, termed “automimicry.” Although females in chemically defended taxa often lay their eggs in clusters, leading to siblings living in close proximity, current models of automimicry have neglected kin‐selection effects, which may be expected to curb the evolution of such selfishness. Here, we develop a general theory of automimicry that explicitly incorporates kin selection. We investigate how female promiscuity modulates intragroup and intragenomic conflicts overinvestment into chemical defense, finding that individuals are favored to invest less than is optimal for their group, and that maternal‐origin genes favor greater investment than do paternal‐origin genes. We translate these conflicts into readily testable predictions concerning gene expression patterns and the phenotypic consequences of genomic perturbations, and discuss how our results may inform gene discovery in relation to economically important agricultural products.

## 1 INTRODUCTION

Risk of predation is a widespread and powerful selective influence across the natural world. Insects and other small invertebrates are often sufficiently chemically defended to deter many predators (Eisner, Eisner, & Siegler, 2005), and this chemical defense is often correlated with aggregation and distinctive aposematic warning signals (Sillen‐Tullburg, 1988; Ruxton & Sherratt, 2006). Aggregation is considered to be an important aspect of chemical defense because there is considerable evidence of a “public good” element (Jones, Speed, & Mappes, 2016). Specifically, aggregation of chemically defended prey is often expected because a predator that experiences an adverse reaction to tasted or ingested chemicals after attacking one individual is less likely to attack similar‐looking neighbors (e.g., Sillén‐Tullberg & Leimar, 1988).

There are a number of mechanisms by which investment in defense might be expensive for the individual, and evidence of such costs abounds (reviewed by Speed, Ruxton, Mappes, & Sherratt, 2012). In situations where costs of defense are paid by the individual, and predators cannot readily identify an individual's level of investment without paying a sampling cost, so at least some of the benefits are available as a common good to the individual's groupmates, one might expect some individuals to “cheat” by not investing in defenses themselves and taking advantage of the investment of those around them. As this phenomenon hinges on the predator's inability to differentiate “cheats” from conspecifics that do invest in defenses, it has been termed “automimicry” (Brower, van Brower, & Corvino, 1967). In fact, it is widely reported that often a nontrivial proportion of an otherwise chemically defended population lacks defensive toxins altogether (Speed et al., 2012). There has been considerable theoretical exploration of the evolution and maintenance of automimicry and prediction of the fraction of automimics in different ecological circumstances (e.g., Brower, Pough, & Meck, 1970; Guilford, 1994; Ruxton & Speed, 2006; Speed, Ruxton, & Broom, 2006; Svennungsen & Holen, 2007).

Current theoretical understanding of automimicry is based on exploration of the direct fitness of aggregating individuals that either do or do not invest in chemical defense. The implicit assumption of this approach is that individuals within an aggregation are genetically unrelated, such that kin‐selection effects can be ignored. However, this is not a good representation of many systems in which automimicry occurs. Specifically, many lepidoptera and other insects with herbivorous larvae that exhibit automimicry are characterized by females laying their eggs in clusters (Ruxton & Sherratt, 2006). Such egg clustering can lead to aggregations of larvae being composed of siblings (Courtney, 1984) and, indeed, this was the basis for Fisher's (1930) hypothesis that antipredator distastefulness evolves via benefits to siblings—an early application of kin‐selection theory, and the first clear, quantitative use of the kin‐selection coefficient of relatedness. Although Fisher assumed full‐sibling broods, females often mate with several males before oviposition (Arnqvist & Nilsson, 2000), yielding a mix of full‐ and half‐siblings within an aggregation (Costa, 2006).

Here, we develop a general theory of automimicry that explicitly considers kin‐selection effects. We use our theoretical framework to investigate how female promiscuity may modulate intragroup and intragenomic conflicts over investment into costly distastefulness, and we translate these results into readily testable predictions concerning patterns of gene expression at loci underpinning distastefulness and the phenotypic consequences of a range of natural or experimentally induced mutations and epimutations. We discuss how these results may inform gene discovery in relation to economically and societally important agricultural products.

## 2 INTRAGROUP CONFLICT

Following Fisher (1930), we consider that a focal individual resides within a large aggregation of siblings, and that if she increases her investment into distastefulness (as a proxy for chemical defense more generally), then this reduces the probability that her siblings are attacked by predators. We generalize upon Fisher's scenario in two ways: first, whereas Fisher assumed that distastefulness was a costless trait, we consider that increased investment into distastefulness may reduce the individual's own fitness; and, second, whereas Fisher implicitly assumed aggregations of full siblings—that is, individuals sharing the same mother and father—we consider aggregations of maternal siblings who may have different fathers.

Using kin‐selection methodology (Bulmer, 1994; Frank, 1998; Pen, 2000; Taylor, 1996; Taylor & Frank, 1996), we are able to show in very general terms that there is a systematic mismatch between the level of investment in distastefulness that maximizes the individual's inclusive fitness (*z*_I_*; Hamilton, 1964) and the level that maximizes the fitness of her group (*z*_G_*; Gardner & Grafen, 2009). Specifically, the focal individual is never favored to invest more into distastefulness than is optimal for her group, and will often be favored to invest considerably less, with her investment monotonically decreasing with the degree of female promiscuity (*z*_I_* ≤ *z*_G_* and d*z*_I_*/d*p* < 0, where *p* is the probability that two maternal siblings have different fathers; see Appendix for derivation).

As a concrete illustration of these results, we consider a scenario in which there is a large number of aggregations with each aggregation containing a large number of maternal siblings. We assume that an individual's probability of survival to adulthood in the face of the threat of predation is an increasing, linear function *S *= *S*_max_ *y* of her group's average investment *y* into distastefulness, where *S*_max_ is the probability of survival if all group members invest all their resources (*y *=* *1) into distastefulness rather than into future reproductive success. And, we assume that conditional upon surviving to adulthood, an individual's expected fecundity is a decreasing, linear function *F *=* F*_max_(1 − *x*) of her own investment *x* into distastefulness, where *F*_max_ is the expected fecundity if the individual invests none of her resources (*x *=* *0) into distastefulness. Upon surviving to adulthood individuals leave their aggregations to mate at random with nonrelatives, then each mated female produces a large number of offspring—aggregated into maternal‐sibling groups—in proportion to her fecundity. All adults then die, returning the population to the beginning of the life cycle.

**Figure 1:**
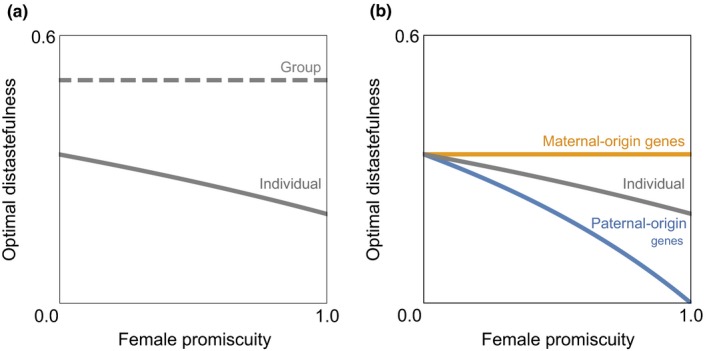
Intragroup and intragenomic conflicts over distastefulness. (a) The level of investment into distastefulness that maximizes the individual's inclusive fitness (*z*_I_*) is always less than that which maximizes the overall fitness of the group (*z*_G_*), and it is a decreasing function of female promiscuity (*p*). (b) The level of investment into distastefulness that maximizes the individual's maternal‐origin genes’ inclusive fitness (*z*_M_*) is always greater than that which maximizes the individual's paternal‐origin genes’ inclusive fitness (*z*_P_*), except for when all maternal siblings are also paternal siblings (*p *=* *0)

With these simplifying, illustrative assumptions, we find that while an equal investment into distastefulness versus fecundity maximizes the overall fitness of the group (*z*_G_* = ½; dashed gray line in Figure 1a), a lower investment into distastefulness is favored by the individual (*z*_I_* = (2 − *p*)/(6 − *p*); solid gray line in Figure 1a). Moreover, the level of investment into distastefulness that maximizes the individual's inclusive fitness is a decreasing function of female promiscuity, with an investment of one‐third being favored in the context of strict female monogamy (*z*_I_* = 1/3 when *p *=* *0), an investment of one‐fifth in the context of extreme female promiscuity (*z*_I_* = 1/5 when *p *=* *1), and an investment of intermediate value in the context of intermediate female promiscuity (1/5 < *z*_I_* < 1/3 when 0 < *p *<* *1; full derivations given in the Appendix).

## 3 INTRAGENOMIC CONFLICT

Natural selection is predicted to adjust the level of investment into distastefulness according to that which maximizes the individual's inclusive fitness (*z*_I_*) when the underlying genes are ignorant of their parent of origin. In the event that parent‐of‐origin information is available to these genes, then we expect that they may come into conflict with each other regards to this trait (cf Burt & Trivers, 2006; Haig, 1996). Specifically, a gene that knows itself to have originated from its carrier's mother is relatively more likely to be carried by maternal siblings who benefit from the individual increasing her investment into distastefulness, and a gene that knows itself to have originated from its carrier's father is relatively less likely to be carried by these maternal siblings as they need not be paternal siblings.

Focusing on the gene's own inclusive‐fitness optimum (Gardner, 2014; Gardner & Welch, 2011), we find that when a gene knows it is of maternal origin, it prefers an individual‐level investment into distastefulness greater than or equal to that which maximizes the individual's inclusive fitness (*z*_M_* ≥ *z*_I_*), and when a gene knows it is of paternal origin, it prefers a level of investment less than or equal to that which maximizes the individual's inclusive fitness (*z*_P_* ≤ *z*_I_*; see Appendix for derivation). Such divergence in the inclusive‐fitness optima of different genes residing in the same genome defines intragenomic conflict (Gardner & Úbeda, 2017).

As an illustration, we consider the simple model described in the previous section, and find that the optimal level of investment into distastefulness from the perspective of a maternal‐origin gene is one‐third irrespective of the degree of female promiscuity (*z*_M_* = 1/3 for all 0 ≤ *p *≤* *1; orange line in Figure 1b), whereas the optimum for a paternal‐origin gene decreases from one‐third to zero as the degree of female promiscuity rises from zero to unity (*z*_P_* = (1 − *p*)/(3 − *p*), and hence *z*_P_* = 1/3 when *p *=* *0 and *z*_P_* = 0 when *p *=* *1; blue line in Figure 1b). Accordingly, there is a discrepancy between the inclusive‐fitness optima of maternal‐origin versus paternal‐origin genes (intragenomic conflict) in the context of female promiscuity (*z*_M_* > *z*_P_* when *p *> 0) but not in the context of strict female monogamy (*z*_M_* = *z*_P_* when *p *=* *0; see Appendix for derivation).

## 4 GENOMIC IMPRINTING

The conflict of interest arising between a maternal‐origin gene and paternal‐origin gene at a locus for which parent‐of‐origin information is available has been suggested to drive the evolution of parent‐of‐origin‐specific gene expression, or “genomic imprinting” (Haig, 2002). According to the “loudest voice prevails” principle (Haig, 1996), if the locus under consideration encodes a gene product that increases the level of a contested trait (a “promoter” locus), then the gene with the larger‐valued optimum is favored to increase its level of expression and the gene with the lower‐valued optimum is favored to decrease its level of expression, resulting in the silencing of the gene with the lower‐valued optimum. Conversely, for a locus that encodes a gene product that decreases the level of the trait (an “inhibitor” locus), similar logic predicts that the gene with the larger‐valued optimum will be silenced.

**Figure 2:**
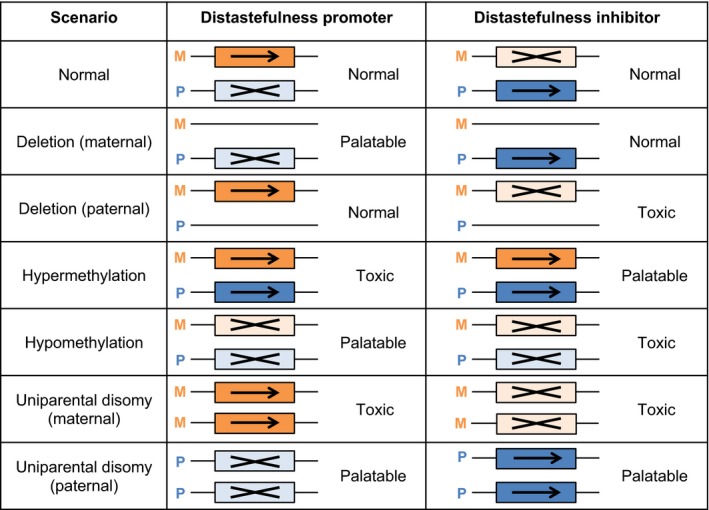
Genomic imprinting and associated patterns of maladaptation. A locus at which the gene product increases distastefulness (a “distastefulness promoter”) is predicted to be maternally expressed and paternally silenced, such that: Deletion of the maternal‐origin gene will lead to underexpression of this product and hence an abnormally low level of distastefulness (a “palatable” phenotype), whereas deletion of the paternal‐origin gene will have no effect (a “normal” phenotype); hypermethylation will activate the normally silenced paternal‐origin gene and hence yield an abnormally high level of distastefulness (“toxic” phenotype), whereas hypomethylation will silence the normally expressed maternal‐origin gene and hence yield a palatable phenotype; both genes being inherited from the individual's mother (“maternal uniparental disomy”)—and hence both being expressed—yields a toxic phenotype, whereas both genes being inherited from the individual's father (“paternal uniparental disomy”)—and hence both being silenced—yields a palatable phenotype. Conversely, a locus at which the gene product decreases distastefulness (a “distastefulness inhibitor”) is predicted to be maternally silenced and paternally expressed, such that: Deletion of the maternal‐origin gene yields a normal phenotype, whereas deletion of the paternal‐origin gene yields a toxic phenotype; hypermethylation yields a palatable phenotype, whereas hypomethylation yields a toxic phenotype; and maternal uniparental disomy yields a toxic phenotype, whereas paternal uniparental disomy yields a palatable phenotype. (Note that, in insects, methylation appears to be associated with increased gene expression, rather than reduced gene expression more commonly observed in vertebrates; Glastad et al., 2014)

Applying the logic of the loudest‐voice‐prevails principle to distastefulness in the context of female promiscuity, then: If the focal locus encodes a gene product that increases distastefulness (a “distastefulness promoter”), we predict that the paternal‐origin gene, having the lower‐valued optimum (*z*_P_*), will be silenced, and the maternal‐origin gene, having the larger‐valued optimum (*z*_M_*), will be expressed; and if the locus encodes a gene product that decreases distastefulness (a “distastefulness inhibitor”), we predict that the maternal‐origin gene, having the larger‐valued optimum (*z*_M_*), will be silenced, and the paternal‐origin gene, having the lower‐valued optimum (*z*_P_*), will be expressed (Figure 2).

These predictions concerning parent‐of‐origin‐specific patterns of gene expression themselves give rise to a suite of predictions concerning how a variety of mutational and epimutational perturbations will affect the distastefulness phenotype (Figure 2). Specifically, we consider: (1) gene deletions or, equivalently, loss‐of‐function point mutations; (2) imprinting disruptions, either in the form of hypermethylation (whereby a gene that is not normally methylated becomes so, which appears to be associated with activation of gene expression in insects; Glastad, Hunt, & Goodisman, 2014) or in the form of hypomethylation (whereby a gene that is normally methylated becomes unmethylated, which appears to be associated with a loss of gene expression); and (3) uniparental disomies (whereby both genes at the focal locus derive from the same parent, rather than one from each parent). Such perturbations may be naturally occurring or experimentally induced, and provide additional avenues for empirical testing.

## 5 DISCUSSION

We have developed the first general theoretical framework for understanding the evolution of costly distastefulness—and other forms of antipredator chemical defense—that incorporates kin‐selection effects, extending Fisher's (1930) initial insight that benefits to siblings may drive the evolution of such traits even if they do not benefit the individual directly. This has enabled us to properly characterize the tension that exists between the interests of individual and group with regard to investment into individually costly but group‐beneficial chemical defenses—with the individual predicted to invest systematically less into chemical defense than is optimal for the group—and how this may be modulated by demographic factors that influence kinship within aggregations, such as female promiscuity. We have framed this tension in terms of “automimicry,” in the sense of individuals deriving a benefit from resemblance to chemically defended conspecifics (Brower et al., 1967), rather than in the alternative sense of one part of an individual resembling a different part of the same individual (Poulton, 1890). Although automimicry may often involve aposematic signaling, our analysis also applies to scenarios in which there is no explicit advertising of distastefulness. The importance of kin grouping to the evolution of aposematic signaling has previously been explored (e.g., Brodie & Agrawal, 2001). We have also investigated an associated intragenomic conflict of interests in which an individual's maternal‐origin genes, being relatively more related to the beneficiaries of group defense, are favored to have the individual invest more in costly chemical defense, than are the individual's paternal‐origin genes, and we have explored how this conflict is expected to shape patterns of gene expression and associated organismal maladaptation, which not only provides avenues for empirically testing the theory but also provides means by which the genes underpinning economically important chemical‐defense phenotypes may be identified.

Our analysis has focused upon how the quantitative level of distastefulness is molded by natural selection in the context of maternal‐sibling aggregations, and applies irrespective of whether the evolution of gregariousness precedes or follows the initial evolution of unpalatability (e.g., Sillén‐Tullberg, 1988; Sillén‐Tullberg & Leimar, 1988). The phenotype we have investigated could represent the actual quantitative investment made by an individual into distastefulness or, alternatively, her probability of developing as a qualitatively distinct, unpalatable morph, and our analysis thereby applies equally to scenarios involving continuous versus discrete individual variation in realized distastefulness. Regarding scenarios in which aggregations comprise individuals of distinct palatable versus unpalatable morphs, the palatable forms have often been conceptualized as “cheats,” free riding on their group's investment into costly defense and enjoying a selfish advantage (Guilford, 1994). However, depending upon how predators respond to prey aggregations in which the same total amount of toxin is concentrated into a few individuals versus more‐or‐less equally produced by all members of the aggregate, the existence of two distinct morphs may instead represent a cooperative division of labor. In such scenarios, cheats would have a greater probability of developing as the palatable morph, but not all palatable individuals would be cheats. Moreover, the unpalatable morph would represent a helper caste which, improving the survival of their siblings at the cost of their own reproductive success, would qualify such species as “eusocial” according to Crespi and Yanega's (1995) definition.

We have shown that when the group benefits of protection against predation accrue to maternal siblings who are not necessarily paternal siblings, an individual's maternal‐origin genes are relatively more favored to have the individual invest in such defenses than are the individual's paternal‐origin genes. Applying the “loudest voice prevails” principle—formulated analytically by Haig (1996) and recently given computer‐simulation support by Farrell, Úbeda, and Gardner (2015)—we have shown that this intragenomic conflict is expected to drive silencing of paternal‐origin genes at loci encoding gene products that promote investment into chemical defenses, and silencing of maternal‐origin genes at loci encoding gene products that inhibit investment into chemical defenses. Accordingly, we have provided a suite of predictions as to how loci underpinning such phenotypes are expected to show parent‐of‐origin‐specific gene expression and how the phenotype will respond to a variety of mutational and epimutational perturbations, including experimentally induced knockout mutations.

Genomic imprinting has traditionally been associated with mammals and flowering plants, and until recently, the prevailing view has been that it is absent from insects, on account of fruit flies lacking key DNA‐methylation enzymes (Yan et al., 2014). However, recent research has revealed extensive methylation across all insect orders within which it has been sought, with the notable exception of flies (Bewick, Vogel, Moore, & Schmitz, 2017). Moreover, predictions of the kinship theory of genomic imprinting have recently been experimentally confirmed in honeybees (Galbraith et al., 2016; Queller, 2003). Furthermore, parent‐of‐origin information is clearly retained postfertilization at the level of whole haploid chromosome sets in the many insect species that exhibit paternal genome elimination (Ferguson‐Smith, 2011; Gardner & Ross, 2014).

Here, we have made predictions about how potential conflict between maternal and paternal genetic inherences might be resolved with respect to chemical defenses of aggregated prey. There are can also be nongenetic maternal and paternal contributions to chemical defense. Dussourd et al. (1988) showed that eggs of a moth (*Utetheisa ornotrix*) are protected from predators by chemicals that can have maternal or paternal origin: The parents sequester these defensive compounds from their host plants when they are larvae, the father passes these compounds to the mother by seminal infusion, and the mother is able to store paternally derived compounds and confer them along with ones she ingested herself; females often mate multiply, and lay eggs in clusters, such that larvae may be protected by chemicals their mother obtained from males other than their father (Bezzerides & Eisener, 2002). Such biparental endowment of defensive chemicals may be quite widespread taxonomically (Camarano, González, & Rossini, 2009), and accordingly understanding the interplay of genetic versus nongenetic contributions to defense from both parents would be a valuable extension to the work presented here.

Our predictions provide new genomics era avenues for testing basic evolutionary ecological theory in relation to classic social evolutionary phenotypes. Indeed, as the predictions have been made in the complete absence of any empirical information regarding the direction or even the existence of genomic imprinting at loci underpinning antipredator chemical defense, there is an opportunity here for a completely independent test of theory, which avoids the circularity encountered when theory is inspired by, and put to the test against, the very same sources of empirical data (Queller, 2003; Queller & Strassmann, 2002; Rautiala & Gardner, 2016; Wild & West, 2009).

Moreover, these predictions may be used to identify candidate genes underpinning chemical‐defense phenotypes, and help assess their function (cf Farrell et al., 2015). For example, we predict that chemical‐defense‐promoter loci at which parent‐of‐origin information is available will be maternally expressed and paternally silenced, and this provides a filter that may greatly narrow the search for candidate loci. Such genes are of basic evolutionary ecological interest, but are often also of strong economic importance. For example, carmine‐based dyes derived from the antipredator chemical defenses of cochineal scale insects have been historically important in the food and textiles industries and are enjoying a resurgence of popularity given the rising demand for natural agricultural alternatives to synthesized chemical products (Greenfield, 2005).

## CONFLICT OF INTEREST

None declared.

## AUTHOR CONTRIBUTIONS

RB, GDR, and AG designed the research. RB and AG performed the analyses. RB, GDR, and AG wrote the manuscript.

## APPENDIX 1 DERIVATION OF MATHEMATICAL RESULTS

### General predictions

An individual's relative, personal fitness may be written as *W*(*x*,*y*,*z*), where *x* is her expected investment into distastefulness, *y* is the average investment made by her group, and *z* is the average investment made across the population. Consider a locus that influences investment into distastefulness and denote by *g* the genic value of a gene drawn at random from the population at this locus. The condition for natural selection to favor an increase in investment into distastefulness is d*W*/d*g* > 0, where *W* is the relative fitness of the individual who carries the gene and where a larger value of g is associated with a greater investment into distastefulness (Taylor, 1996). Assuming that a gene's phenotypic effect does not depend upon its parent of origin, this derivative may be expressed as dW/dg=(∂W/∂x)×(dx/dG)× $\left(dG/dg\right)+\left(\partial W/\partial y\right)\times \left(dy/dG^{\prime }\right)\times \left(dG^{\prime }/dg\right)$, where *G* is the individual's genetic breeding value for distastefulness, *G*′ is the average breeding value in the individual's group, d*x*/d*G* = d*y*/d*G*′ = γ describes the map between genotype and phenotype, d*G*/d*g* = *q* describes the consanguinity of the individual to itself, d*G*′/d*g* = *q*′ describes the consanguinity of the individual to a random member of her group and all derivatives are evaluated at *x *= *y *= *z* (Frank, 1998; Taylor, 1996; Taylor & Frank, 1996). Making these substitutions into the condition for increase obtains Hamilton's (1964) rule −*C*(*z*) + *B*(*z*)*r *>* *0, where −*C*(*z*) = ∂*W*/∂*x*,* B*(*z*) = ∂*W*/∂*y* and *r *= *q*′/*q* is the kin‐selection coefficient of relatedness (Bulmer, 1994). Note that individual and group interests exactly coincide when *r *=* *1 (Gardner & Grafen 2009), such that an increase in investment into distastefulness is favored from the group's perspective if −*C*(*z*) + *B*(*z*) > 0.

Relaxing the assumption that gene effects are independent of parent of origin, we now consider the interests of maternal‐origin versus paternal‐origin genes by granting full control of the distastefulness phenotype to each party in turn and seeing what levels of investment into distastefulness each is favored to bring about. First, granting control to the individual's maternal‐origin gene at the focal locus, the action of natural selection is given by $dW/dg=\left(\partial W/\partial x\right)\times \left(dx/dG_{M}\right)\times \left(dG_{M}/dg\right)+\left(\partial W/\partial y\right)\times \left(dy/dG_{M}^{\prime }\right)\times \left(dG_{M}^{\prime }/dg\right)$, where *G*_M_ is the individual's maternal‐origin genetic breeding value for distastefulness, *G*′_M_ is the average maternal‐origin genetic breeding value in the individual's group, d*x*/d*G*_M_ = d*y*/d*G*′_M_ = γ_M_ describes the map between maternal‐origin genotype and phenotype, d*G*_M_/d*g* = *q*_M_ **= *q* describes the consanguinity of the individual's maternal‐origin gene to the individual itself, d*G*′_M_/d*g* = *q*′_M_ describes the consanguinity of an individual's group members’ maternal‐origin genes to the individual itself, and all derivatives are evaluated at *x *= *y *= *z*. This yields the condition for increase −*C*(*z*) + *B*(*z*)*r*_M_ > 0, where *r*_M_ = *q*′_M_/*q*. Similar logic yields the condition −*C*(*z*) + *B*(*z*)*r*_P_ > 0 when full control of the phenotype is given to the individual's paternal‐origin gene.

In general, then, the action of natural selection is given by −*C*(*z*) + *B*(*z*)ρ, where ρ is the kin‐selection coefficient of relatedness appropriate to the controller of the distastefulness trait. We may define a function *J*(*z**, ρ) = −*C*(*z**) + *B*(*z**)ρ where *z** represents a non‐boundary convergence stable evolutionary equilibrium and hence satisfies *J*(*z**, ρ) = 0 and ∂*J*/∂*z** < 0 (Taylor, 1996). Consequently, we may write $dJ/d\rho =\partial J/\partial \rho +\partial J/\partial z^{*}\times dz^{*}/d\rho =0$, which rearranges as d*z**/dρ = −(∂*J*/∂ρ)/(∂*J*/∂*z**) and hence reveals that $\text{Sign}(dz^{*}/d\rho )=$ $\text{Sign}\left(\partial J/\partial \rho \right)=\text{Sign}(B\left(z^{*}\right))$ (Pen, 2000). That is, if such a convergence stable equilibrium exists, it is a monotonically increasing function of ρ so long as increasing investment in distastefulness improves the fitness of one's group mates (*B *>* *0). This means that so long as an individual is more related to its group mates via its maternal‐origin genes than via its paternal‐origin genes (*r*_M_ > *r*_P_), then its maternal‐origin genes favor larger investment in distastefulness than do its paternal‐origin genes (*z*_M_* > *z*_P_*).

### Illustrative model

Adopting the assumptions of the illustrative model presented in the main text, we have fitness given by *w*_f_(*x*,*y*) = *S*_max_ *y *× *F*_max_(1 − *x*) for females and hence relative female fitness given by *W*_f_(*x*,* y*,* z*) = *w*_f_(*x*,* y*)/*w*_f_(*z*,* z*) = (*y*/*z*)(1 − *x*)/(1 − *z*). Similarly, male fitness is given by *w*_m_(*x*,*y*) = *S*_max_ *y *× [(1 − *x*)/(1 − *z*)] and relative male fitness is given by *W*_m_(*x*,*y*,*z*) = (*y*/*z*)(1 − *x*)/(1 − *z*). Accordingly, *W *= ½ *W*_f_ + ½ *W*_m_ (Taylor & Frank, 1996), and a convergence stable evolutionary equilibrium satisfies −*C*(*z**) + *B*(*z**)ρ = 0, where $-C\left(z^{*}\right)=\partial W_{f}/\partial x{{|}_{x=y=z=z^{*}}=\partial W_{m}/\partial x|}_{x=y=z=z^{*}}=$ $-1/(1-z^{*})$, $B\left(z^{*}\right)=\partial W_{f}/\partial y{{|}_{x=y=z=z^{*}}=\partial W_{m}/\partial y|}_{x=y=z=z^{*}}=1/z^{*}$ and ρ* *= *r*_M_ for genes that know they are of maternal‐origin, ρ* *= *r*_P_ for genes that know they are of paternal‐origin, and ρ* *= *r* for genes that are ignorant of their parent of origin. Drawing two genes at random from the same locus from oneself, the probability that they are identical by descent is *q *= ½. Drawing a gene at random from ones groupmate, the probability it is identical by descent to a gene drawn at random from oneself is *q*′ = ¼ × *q *+ ½ × 0 + ¼ × (1 − *p*) × *q*, because with probability ¼, both genes are maternal in origin and as group mates are maternal siblings, the resulting consanguinity is *q*, with probability ½, one gene is maternal in origin and the other is paternal in origin and because mating partners are unrelated, the resulting consanguinity is 0, and with probability ¼, both genes are paternal in origin and hence with probability 1 − *p,* the two group mates are paternal siblings and the resulting consanguinity is *q*. Drawing a gene at random from a group mate, then the probability that it is identical by descent with ones own maternal‐origin gene residing at that locus is *q*′_M_ = ½ × *q *+ ½ × 0, and the probability that it is identical by descent with ones own paternal‐origin gene residing at that locus is *q*′_P_ = ½ × 0 + ½ × (1 − *p*) × *q*. This yields *r *= *q*′/*q *= (2 − *p*)/4, *r*_M_ = *q*′_M_/*q *= ½ and *r*_P_ = *q*′_P_ = (1 − *p*)/2, and hence *z*_I_* = (2 − *p*)/(6 − *p*), *z*_M_* = 1/3, *z*_P_* = (1 − *p*)/(3 − *p*) and *z*_G_* = 1/2, as reported in the main text.
